# Impact of being small for gestational age in neonates born below 600 g birth weight

**DOI:** 10.1038/s41390-025-04202-x

**Published:** 2025-06-23

**Authors:** Raphaela Jernej, Renate Fuiko, Julia Binder, Herbert Kiss, Katrin Klebermass-Schrehof, Agnes Grill, Angelika Berger, Katharina Goeral

**Affiliations:** 1 Comprehensive Center for Pediatrics, full Member of ERN EpiCARE, Department of Pediatrics and Adolescent Medicine, Division of Neonatology, Intensive Care and Neuropediatrics, Medical University of Vienna, Vienna, Austria; 2 Comprehensive Center for Pediatrics, Department of Obstetrics and Gynecology, Division of Obstetrics and fetomaternal Medicine, Medical University of Vienna, Vienna, Austria

## Abstract

**Background:**

There are controversial data regarding the impact of being small for gestational age (SGA) on outcome of infants born below 600 g. Comparing mortality, morbidities and neurodevelopmental outcome at 2-3 years in GA-matched very low birth weight neonates born SGA vs. appropriate for gestational age (AGA).

**Methods:**

Retrospective single centre matched cohort study including infants born ≥22 weeks GA 2012–2022. Study group was defined as ≤600 g birthweight and SGA and matched with AGA controls for GA, sex, and year of birth.

**Results:**

A total of 103 SGA and 103 AGA infants were matched. SGA neonates had higher rates of surfactant administration, surgically treated persistent ductus arteriosus, bronchopulmonary dysplasia, and retinopathy of prematurity intervention. Survival until discharge was 56% in SGA and 85% in AGA neonates. Using Bayley Scales of Infant Development at 2–3 years 42% of SGA and 66% of AGA infants showed normal cognitive outcome, 45% SGA and 60% AGA infants had normal motor development. Severe neurodevelopmental impairment was present in 10 SGA (26%) and 12 AGA survivors (18%, *p* = 0.102).

**Conclusion:**

SGA patients exhibited significantly higher mortality rates and increased incidences of short-term morbidities compared to AGA neonates. Significant difference regarding cognitive and motor development was observed.

**Impact:**

- There is a lack of studies examining the differences between SGA and AGA infants, particularly within the very low birth weight (VLBW) category. Despite this gap, SGA infants continue to face notable disadvantages.
- This paper contributes novel insights into the outcomes of VLBW SGA patients. Survival rate was 56% in SGA compared to 85% in AGA neonates. A total of 26% of SGA and 18% of AGA infants experienced severe neurodevelopmental impairment.
- The study’s findings may pave the way for improved parental counselling, by providing valuable centre-based data to ensure informed decision-making and support families more effectively in the future.

## Introduction

Several studies demonstrated an increased risk of mortality, severe morbidities, as well as adverse neurodevelopmental outcome in neonates with a very low birthweight (VLBW).^1,2^ However, there is a limited number of studies comparing SGA and appropriate for gestational age (AGA) infants within the group of VLBW infants. About 16% of all neonates worldwide are SGA.^3^ There is a variety of definitions of small for gestational age (SGA) infants including birthweight <2 SDs from the median or, birthweight <5^th^ or <10^th^ percentile of the reference.^3^ The aetiology is various and complex and includes maternal, placental, foetal and genetic factors.^4^ Both SGA and foetal growth restriction (FGR) are associated with preterm delivery,^5,6^ which may lead to prematurity-associated morbidities. Despite advances in perinatal medicine reducing the overall risk of mortality and morbidities in recent decades, SGA infants continue to face greater risks compared to their appropriate for gestational age (AGA) counterparts.^7^ Most available studies focused on infants <1500 g birthweight or up to 37 weeks gestational age (GA).^8,9^

We previously explored mortality, short-term morbidities and long-term neurological outcomes in infants born <500 g and highlighted that many of these neonates experience at least one severe short-term morbidity. However, more than one-third of studied patients did not show severe neurodevelopmental impairment at two years corrected age.^10^ In this study we further investigate the cohort of VLBW neonates, especially those with a birthweight ≤600 g, by comparing differences between matched SGA and AGA infants. We expanded the cohort of the most vulnerable neonates using this specific cut-off because we observed better outcomes in those born >600 g at our centre, while also aiming to analyse a larger and more statistically significant cohort compared to our previous publication.^10^ The aim was to generate centre-based data for parental counselling and evaluate potential disadvantages faced when being SGA within the cohort of VLBW neonates treated in an academic tertiary perinatal centre.

## Patients and methods

This retrospective study was conducted at our tertiary perinatal centre, the Medical University of Vienna, Austria, with 54 beds specifically dedicated to neonatal care. The primary outcome was mortality until discharge. Secondary outcomes included morbidity rates until discharge and neurodevelopmental outcome assessed at 2–3 years of age.

Inborn preterm neonates born between May 2012 and December 2022 were included. During this period, an average of 2534 babies were born in our hospital per year. Interdisciplinary antenatal counselling, involving neonatologists, obstetricians and psychologists, is provided for births <28 weeks GA, along with a proactive approach beginning at 23 weeks GA.^11^ Our postnatal delivery room management for neonates born <28 weeks GA consists of plastic bags to prevent hypothermia, caffeine-citrate, high-flow Jet-CPAP via Benveniste Valve^TM^ (Dameca, Löwenstein Group, Rødovre, Denmark) via facemask and early prophylactic less invasive surfactant administration (LISA).^11,12^

The study group consisted of SGA neonates with a birthweight ≤600 g and <10th percentile for GA according to sex specific standards based on the Fenton preterm growth chart 2013.^13^ Controls (AGA group) were matched at a ratio of 1:1 with the SGA group based on GA, sex and year of birth, and were required to have a birthweight between the 25^th^ and 75^th^ percentile for GA. We primarily matched based on the nearest GA day, and secondly by selecting the neonate closest to the 50th birth weight percentile. The exclusion criteria were GA < 22 + 0, prepartum determined comfort care, major congenital and chromosomal anomalies.

Both neonatal and maternal data were collected from electronic patient records. Gestational age was defined either by crown-rump length measured at first trimester screening or by the last menstrual period. FGR was defined as intrauterine weight <10th percentile and/or sudden reduction in the expected foetal growth pattern during pregnancy in addition to pathological doppler sonography and/or oligohydramnios, based on the definition of Aplin et al.^14^ The impact of FGR on both short- and long-term outcome was assessed only in SGA neonates, as the number of FGR cases among the AGA group was only two. At our unit, enteral nutrition is started on the first day of life with mother’s milk or pasteurized preterm single donor milk, or preterm formula for infants born >32 weeks of gestation. Enteral feedings are fortified once an enteral intake of 100 mL/kg/day is reached. Parenteral nutrition is also initiated from day one of life and continued until an enteral intake of around 130–150 mL/kg/day is achieved. Weight is measured every 48 hours, and length and head circumference weekly. Regarding early intervention services, all preterm infants receive physical and occupational therapy during their inpatient stay, with speech therapy provided when needed. We also offer nutritional and lactation counselling.

Post-discharge, we follow a standardized developmental monitoring programme with follow-up examinations until 5.5 years of life, which includes assessment of cognitive, motor and language skills, as well as growth. This ensures the early initiation of further interventions if necessary.

While weight is monitored and considered an important growth parameter, discharge readiness in our unit was primarily based on factors such as cardiorespiratory stability without respiratory support, temperature stability without external heat supply, feeding success and adequate weight gain relative to extrauterine growth percentiles and growth trajectory. These criteria may influence the observed growth patterns, as neonates who are otherwise clinically stable may still exhibit disproportionate growth trajectories by the time of discharge.

### Short-term outcome

Several neonatal morbidities were studied: “Severe brain injury^15^” was defined as intraventricular haemorrhage (IVH) grade ≥3 and/or cystic periventricular leukomalacia (cPVL).^16^ Persistent ductus arteriosus (PDA) was defined as a hemodynamically significant duct, determined by clinical signs and functional echocardiography, that remained open despite three cycles of ibuprofen followed by one cycle of paracetamol, and subsequently required surgical closure by ligation, in accordance with the standard protocol at our centre. Necrotizing enterocolitis (NEC) was defined as NEC stage ≥2 according to Bell,^17^ bronchopulmonary dysplasia (BPD) was defined as oxygen requirement at 36 weeks corrected age and severe retinopathy of prematurity (ROP) as ROP stage ≥3.^18^ requiring intervention (Ranibizumab or laser). “Severe morbidity” was defined as described previously.^10^ as either IVH grade ≥3, cPVL, BPD or severe ROP requiring intervention.

### Long-term outcome

Neurodevelopmental outcome was obtained at 2–3 years via Bayley Scales of Infant Development 3rd edition (Bayley-III) by trained clinical psychologists. Cognitive, Language and Motor Composite Scores were acquired using both German and American norms. As previously described, American norms tend to overestimate neurodevelopmental outcome in Austrian preterms.^19^ However, they were included in our analysis to facilitate comparison with other studies and populations using American norms, thereby providing broader context for our findings. Using German norms, outcome data was classified into four groups using conventional SD banded cut-off points.^20^: normal ≥85, mild impairment 70-84, moderate impairment 55–69, and severe impairment <55. In addition, data regarding cerebral palsy (CP), visual and hearing ability was collected. Finally, the following definitions were used: “favourable outcome”—normal development or mild impairment in all subtests; “unfavourable outcome”—moderate or severe impairment in any subtest.

### Statistical analysis and ethics

For statistical analysis SPSS Statistics, version 27 for Mac (IBM Corporation, Armonk, New York) and GraphPad Prism 6 (GraphPad software, San Diego, California) were used. Demographic data are shown as means ± SD or median and IQR for quantitative data and counts and percentages for qualitative data. A *p* value of <0.05 was considered statistically significant.

The study protocol was approved by the Institutional Review Board of the Medical University Vienna (EK 2098/2022).

## Results

At our centre, 95% (=186 patients) of all live births ≤600 g birthweight were immediately admitted to the neonatal intensive care unit (NICU) during the study-period, including those who died after unsuccessful resuscitation efforts shortly after birth. Of them 60% (*n* = 112) had a birthweight <10th percentile. Only 5% received comfort care. Of those patients, seven were excluded based on mentioned exclusion criteria and two neonates could not be matched, resulting in the final study cohort of 103 SGA patients. Detailed antenatal characteristics of both groups are shown in Table 1.

**Table 1:** Antenatal data

	SGA GROUP *n* = 103	AGA GROUP *n* = 103	*p* value
maternal age at birth (years) ^a^	31.9 (27.3–35.6)	33.1 (29.0–37.5)	0.211
hypertensive disorder of pregnancy	12 (12%)	0 (0%)	**<0.001**
placental insufficiency	8 (11%)	3 (3%)	**0.035**
placental abruption	2 (3%)	4 (4%)	0.651
foetal distress	35 (47%)	13 (13%)	**<0.001**
suspected chorioamnionitis	1 (1%)	10 (10%)	**0.020**
cervical insufficiency	4 (5%)	27 (27%)	**<0.001**
pPROM	11 (11%)	54 (53%)	**<0.001**
days before birth ^a^	16 (8-21)	3 (1-11)	**0.007**
GA at pPROM (weeks) ^a^	22.5 (21.6–23.5)	24.6 (23.7–25.7)	**0.001**
Antenatal steroids			
no	1 (1%)	2 (2%)	0.447
complete	76 (74%)	68 (66%)	
multiples	21 (20%)	21 (20%)	0.810
FGR	74 (73%)	2 (2%)	**<0.001**
c-section	99 (96%)	82 (80%)	**<0.001**

Data are shown as n (%), or median (IQR) ^a^ ; *p* values were calculated using Mann–Whitney U test and chi-square-test.

*SGA* small for gestational age, *AGA* appropriate for gestational age, *GA* gestational age, *pPROM* preterm premature rupture of the membrane, *FGR* foetal growth restriction.

In the SGA group, median GA at birth was 25 + 5 weeks, with a median birthweight of 500 g, compared to a median GA of 25 + 5 weeks and median birthweight of 800 g in the AGA group (Table 2). No clinically significant differences were observed in APGAR scores between the groups.

**Table 2:** Neonatal Data and Short-term outcome in survivors

Neonatal data	SGA GROUP *n* = 103	AGA GROUP *n* = 103	*p* value
male	49 (48%)	53 (52%)	0.577
GA at birth (weeks) ^b^	25.7 (22.9–31.1)	25.7 (22.6-31.1)	0.915
birthweight GA 22+	430 (–)	510 (–)	**<0.001**
birthweight GA 23+	420 (350–490)	600 (540–650)	
birthweight GA 24+	490 (318–540)	651 (560–713)	
birthweight GA 25+	493 (340–593)	795 (640–830)	
birthweight GA 26+	550 (410–600)	843 (750–1030)	
birthweight GA 27+	530 (470–600)	940 (840–1150)	
birthweight GA 28+	555 (470–600)	1095 (1040–1200)	
birthweight GA 29+	560 (440–600)	1280 (1235–1390)	
birthweight percentile ^a^	3 (1–6)	49 (37–58)	**<0.001**
birth length percentile ^a^	3 (0–15)	57 (35–76)	**<0.001**
head circumference percentile ^a^	8 (2–19)	50 (35–69)	**<0.001**
umbilical artery pH ^a^	7.28 (7.20–7.33)	7.30 (7.18-7.36)	0.521
surfactant received ^c^	101 (98%)	92 (89%)	**0.010**
Survival until discharge	58 (56%)	88 (85%)	**<0.001**
**Morbidities in survivors**	**SGA GROUP** ***n*** = **58**	**AGA GROUP** ***n*** = **88**	***p*** **value**
severe brain injury	2 (4%)	8 (9%)	0.195
IVH ≥ grade III	2 (4%)	7 (8%)	0.278
cystic PVL	0 (0%)	2 (2%)	0.252
PDA with surgical intervention	10 (18%)	4 (5%)	**0.010**
NEC with surgical intervention	3 (5%)	4 (5%)	0.844
BPD	30 (56%)	22 (31%)	**0.006**
ROP with intervention	10 (18%)	3 (3%)	**0.004**
**Discharge in detail**			
day of life	99 (85–121)	83 (64–99)	**<0.001**
GA	40.4 (38.3–43.7)	38.1 (36.3–39.6)	**<0.001**
weight (grams)	2430 (2095–3125)	2670 (2264–3020)	0.382
length (cm)	44.0 (41.0–47.5)	45.3 (43.3–48.0)	**0.009**
head circumference (cm)	32.4 (30.4–33.5)	32.0 (31.0–33.6)	0.651
weight percentile	1 (1–5)	21 (9–38)	**<0.001**
length percentile	0 (0–2)	10 (3–27)	**<0.001**
head circumference percentile	1 (1–5)	18 (6–36)	**<0.001**

*SGA* small for gestational age, *AGA* appropriate for gestational age, *BPD* bronchopulmonary dysplasia, *GA* gestational age, *IVH* intraventricular haemorrhage, *NEC* necrotising enterocolitis, *PDA* persistent ductus arteriosus, *PVL* periventricular leukomalacia, *ROP* retinopathy of prematurity.

*p* values were calculated using Mann–Whitney U test and chi-square-test.

Data are shown as *n* (%), median (IQR)^a^ or median (range)^b^

^c^Infants who did not receive surfactant either died before administration (2 SGA, 2 AGA), or were born >28 weeks (9 AGA).

Regarding obstetric data (Table 1), AGA neonates more frequently experienced preterm premature rupture of membranes (pPROM; *p* < 0.001), with a shorter latency between pPROM and birth (*p* = 0.007) compared to the SGA group. AGA neonates also had higher rates of suspected chorioamnionitis (*p* = 0.020) and cervical insufficiency (*p* < 0.001). In contrast, mothers of SGA neonates had a higher incidence of hypertensive disorder of pregnancy (*p* < 0.001), foetal distress (*p* < 0.001), and placental insufficiency (*p* = 0.035). Additionally, 96% of the SGA group were delivered by c-section, compared to 80% in the AGA group (*p* = <0.001).

A total of 56% (58/103) of SGA neonates survived until discharge, compared to 85% (88/103) of AGA neonates (*p* < 0.001; Table 1). In the AGA group, survival rate was 43% (6/14) for neonates with 501-600 g birthweight. In this subgroup, median GA was 26 + 2 weeks in SGA vs. 23 + 4 weeks in AGA neonates (*p* < 0.001, Supplementary Table S1).

Overall, of the 74 SGA neonates (73%) who met the criteria for FGR 50% (37/74) survived until discharge (Supplementary Table S1).

### Short-term outcome

Details on short-term morbidities are shown in Table 2. Significant differences were found, with AGA neonates having a lower incidence of several conditions, including PDA with need of surgical ligation, BPD, and ROP with need of intervention. Among the SGA group, FGR neonates did not show a higher risk for short-term morbidities compared to non-FGR neonates (data not shown).

Additionally, there were significant differences in all growth percentiles (weight-, length- and head circumference) as well as day of life and GA at discharge, with SGA neonates being discharged about two weeks later.

### Long-term outcome

Neurodevelopmental outcome was available in 66% of SGA and 76% of AGA survivors, as shown in Table 3. There was a significant difference in both cognitive and motor outcomes, and a trend regarding language outcome. Cognitive development was favourable in 68% of SGA and 81% of AGA patients. Language development was favourable in 58% of SGA and 73% of AGA patients. Motor development was favourable in 76% of SGA and 87% of AGA patients.

**Table 3:** Neurodevelopmental outcome (Bayley-III) in survivors (*Composite Scores*)

	SGA GROUP *n* = 38	AGA GROUP *n* = 67	*p* value
**Age at testing (months)**	33.0 (32.1–33.8)	33.3 (28.5–33.7)	0.824
**Cognition German**	78 ± 15.8	91 ± 20.5	**0.005**
favourable outcome	26 (68%)	54 (81%)	0.118
**Language German**	74 ± 20.4	83 ± 23.2	0.051
favourable outcome	22 (58%)	49 (73%)	0.120
**Motor German**	77 ± 16.9	85 ± 17.6	**0.014**
favourable outcome	29 (76%)	58 (87%)	0.180
**Cognition American**	93 (80–104)	103 (93–110)	**0.014**
**Language American**	89 (71–97)	95 (82–103)	0.084
**Motor American**	91 (81–99)	97 (90–104)	**0.030**
**Outcome classification - German**			
normal outcome	10 (26%)	24 (36%)	0.102
mild impairment	10 (26%)	26 (39%)	
moderate impairment	8 (21%)	5 (7%)	
severe impairment	10 (26%)	12 (18%)	
favourable outcome ^a^	20 (53%)	50 (75%)	**0.022**

Data are shown as *n* (%), median (IQR); Neurodevelopmental data is based on German norms.

*GMFCS* Gross Motor Function Classification System.

^a^Normal outcome and mild impairment in all subtests.

Regarding visual impairment 7 SGA (18%) and 10 AGA (15%) patients had myopia at 2–3 years needing corrective lenses. No patient in either group developed hearing impairment. CP was present in seven patients, 1 SGA (3%) and 6 AGA (10%). However, only one AGA patient was classified as GMFCS level 3; all others presented with ambulant CP (level ≤2).

Within FGR patients, 39% (15/38) had favourable outcome and 15% presented with severe impairment. There was no significant difference between FGR and non-FGR within the SGA group regarding outcome categories.

## Discussion

Less than 1% of all births at our tertiary perinatal centre are born ≤600 g birthweight and among these, approximately 60% (about 10 neonates/year) are also classified as SGA. Parental counselling as well as outcome prediction remains challenging due to the limited numbers of patients and lack of data. We set out to generate centre-based data in our academic tertiary perinatal centre.

In our study group, the survival rate was 56%, whereas the control group exhibited a notably higher survival rate of 85%, highlighting a significantly increased mortality rate among VLBW SGA neonates. Survival rates in the literature referring to VLBW infants range from 38 to 75%.^2,21^ Focusing on SGA, survival rates span from 0 to 90% depending on GA at birth,^22–24^ though some of the studies included patient >1500 g birthweight. When combining SGA and VLBW infants, survival rates range from 21 to 82%.^8,25,26^ In the majority of previous studies, differences in mortality between SGA and AGA patients were significant,^8,23^ except for Bardin et al. who only included neonates born at 24-26 weeks GA^27^ and Charles et al. who excluded infants who died in delivery room.^22^ Similar to our data, most studies offered intensive care starting at 23 weeks GA, except for Charles et al. who also included infants born at 22 weeks GA.^22^

In contrast to us, some medical centres initially stabilize neonates in the delivery room, admitting only those who can be stabilized to the NICU. This practice varies, and some studies lack clarity on whether deaths in the delivery room are included or excluded in the data, leading to potential selection bias in reported survival rates.^28^

Our mortality rate is gradually decreasing with increasing birthweight, which is consistent with previous studies.^21^ Within our cohort, when specifically examining neonates with birthweights between 501 and 600 g—a group that includes both SGA and AGA patients, survival rate was 34% higher in SGA compared to AGA patients. This disparity is likely due to a 2.7-week lower GA in the AGA subgroup. This indicates that for neonates within a certain birthweight range, GA at birth may be a more important factor influencing survival until discharge than being SGA.

Survival rates for SGA neonates in general, but especially for those with an extremely low birthweight (ELBW), are steadily increasing due to improvements in perinatal and neonatal medicine.^25^ Nevertheless, this cohort of vulnerable neonates still frequently suffers from major morbidities and long-term neurodevelopment is still not improving to the same extend.

In our cohort, 62% of SGA and 53% of GA-matched AGA survivor experienced at least one severe short-term morbidity. SGA neonates were diagnosed significantly more often with BPD, which is in line with previous studies,^22^ and the observation of a decreasing risk for BPD as birthweight percentile increases.^29^ In our study, we did not find significant differences in BPD and ROP rates between SGA neonates with and without FGR, likely due to the small sample size. This limits our ability to draw definitive conclusion regarding the specific vascular impact of FGR in this cohort. However, existing literature supports the hypothesis that FGR may impair vascular development, including a reduction in alveolar number and a decrease in vascular endothelial growth factor (VEGGF) expression, as a result of intrauterine hypoxia.^30,31^ These factors could contribute to the increased risk of BPD and ROP observed in SGA neonates, as FGR has been associated with structural changes in the lungs and retina.^31–33^ While our data did not confirm these findings, the potential for underlying vascular mechanisms remains a plausible explanation for the higher rates of these morbidities in SGA neonates. Future studies with larger sample sizes are required to more clearly differentiate between the outcomes of SGA neonates with and without FGR and to explore the specific contributions of FGR to neonatal morbidities like BPD and ROP.

Based on our findings, both groups showed low rates of severe brain injury (severe IVH: 4% SGA, 8% AGA; cPVL: 0% SGA, 2% AGA). Percentages of severe IVH range from 10–16% in SGA and 6–37% in AGA infants in the literature.^8,25,26,29^

Although we did not find a significant difference in weight-, length-, and head circumference percentiles at discharge, SGA neonates appear to gain weight and head circumference to a greater extent than length during their hospital stay. This discrepancy likely reflects the priority placed on achieving weight gain in the neonatal period, as weight is often viewed as a marker of adequate nutritional support and readiness for discharge. Additionally, the focus of parenteral and enteral nutrition for preterms, particularly those <1000 g, is primarily on protein, fat, and glucose intake in most specialized centres. While this approach effectively supports caloric intake and promotes weight and head circumference growth, it may inherently place less emphasis on nutrients critical for linear growth, which might require more sustained supplementation. This highlights potential challenges in promoting proportional growth, particularly in linear growth, which may require longer-term interventions. Further studies analysing how to promote linear growth in ELBW neonates during hospitalization and post-discharge would be beneficial.

Follow-up assessment at 2–3 years was available in a high percentage of survivors in both groups with the majority showing normal outcome or mild neurodevelopmental impairment in all three subtests, aligning closely with data in the literature.^1,34^ Regarding cognitive outcome, our results align to some extent with previously published studies, reporting Bayley-III mild cognitive impairment or worse in VLBW children ranging from 7 to 36%.^21,35^ In terms of motor outcome, our study shows similar significance to previous findings,^36^ yet we observed a higher rate of normal development compared to other studies.^34^ Respectively, nearly 60% of SGA and 64% of GA-matched AGA patients in our study had favourable outcome when evaluated using German norms. Additionally, we analysed our Bayley III data using American norms and found no significant differences in cognitive and motor outcomes for SGA patients compared to the original analysis. As shown previously, median scores were 9–14 points higher using American norms.^10,19^

We did not find any significant difference in composite outcome classification (combining cognition, language and motor domains) between SGA and AGA groups (Table 3), which is consistent with previous data.^34,37^ However, examining individual domains reveals significant differences, underscoring the importance of evaluating specific neurodevelopmental aspects separately.

### Strength and limitations

Our study is the first focusing on mortality, morbidity and long-term outcome of SGA neonates born below 600 g, hence addressing two high risk conditions simultaneously. We included a large control group matched for GA, sex, and year of birth to reduce selection bias. A comparatively high follow-up rate is another strength of our study. Nevertheless, the gap in age (2–3 years) in our follow-up analysis should be noted as a limitation and was partly caused by the Covid-19 pandemic. Another limitation of this study is, that we inferred neurodevelopment only based on birth weight, without accounting for other important factors such as socioeconomic status, family support, physical, occupational, or speech therapy. Future studies should aim to expand patient number through multicentre collaborations to increase generalizability. Also, long-term neurodevelopmental outcomes at five years and beyond should be explored to provide more robust results.

## Conclusion

Infants born ≤600 g birthweight who are also SGA are rare, even in centres with high birth rates, yet they have a large impact on mortality-rates. In our study, mortality rate was significantly higher in SGA vs. GA-matched AGA neonates. Regarding severe morbidities, SGA infants had a significantly higher rate of PDA with surgical ligation, BPD, and ROP with intervention. Among survivors, a significant difference in terms of cognitive and motor development was observed at 2–3 years. Nevertheless, it is essential to recognize that other factors such as family support, follow-up care, physical and speech therapy, and other early intervention services have a significant impact on the neurodevelopmental outcome of preterm and ELBW neonates.

## Supplementary information

Supplementary Information

## Data Availability

The datasets generated during and/or analysed during the current study are available from the corresponding author on reasonable request.
