# Supplementary material for: Impact of being small for gestational age in neonates born below 600 g birth weight
Source: Pediatr Res. 2025 Jun 23;99(2):567–72. doi: 10.1038/s41390-025-04202-x (PMC12956549; doi:10.1038/s41390-025-04202-x)
Supplement: Supplementary file 1 — Supplementary Information [file 41390_2025_4202_MOESM1_ESM.pdf]

SUPPLEMENTARY TABLE S1. Survival subclasses

	SGA GROUP	AGA GROUP	*p-value*
GA 22+ (n=1)	0 (0%)	1 (100%)	–
GA 23+ (n=13)	3 (23%)	7 (54%)	0.107
GA 24+ (n=20)	11 (55%)	17 (85%)	**0.038**
GA 25+ (n=22)	12 (55%)	17 (81%)	0.065
GA 26+ (n=22)	14 (67%)	21 (96%)	**0.015**
GA 27+ (n=9)	6 (67%)	9 (100%)	0.058
GA 28+ (n=10)	7 (70%)	10 (100%)	0.060
GA 29+ (n=6)	4 (67%)	6 (100%)	0.121
<400g (n=13/0)	1 (8%)	0 (0%)	–
401-500 g (n=42/0)	20 (48%)	0 (0%)	–
501-600 g (n=48/14)	37 (77%)	6 (43%)	**0.015**
601-700 g (n=0/21)	0 (0%)	18 (86%)	–
701-800 g (n=0/19)	0 (0%)	19 (100%)	–
801-900 g (n=0/19)	0 (0%)	15 (79%)	–
901-1000 g (n=0/8)	0 (0%)	8 (100%)	–
>1000 g (n=0/22)	0 (0%)	22 (100%)	–

Data are shown as n (%); p-values were calculated using Mann-Whitney U test and chi-square-test.

Abbreviations: SGA, small for gestational age; AGA, appropriate for gestational age; GA, gestational age
